# Web-Based Content on Diet and Nutrition Written in Japanese: Infodemiology Study Based on Google Trends and Google Search

**DOI:** 10.2196/47101

**Published:** 2023-11-16

**Authors:** Kentaro Murakami, Nana Shinozaki, Nana Kimoto, Hiroko Onodera, Fumi Oono, Tracy A McCaffrey, M Barbara E Livingstone, Tsuyoshi Okuhara, Mai Matsumoto, Ryoko Katagiri, Erika Ota, Tsuyoshi Chiba, Yuki Nishida, Satoshi Sasaki

**Affiliations:** 1 Department of Social and Preventive Epidemiology, School of Public Health, The University of Tokyo Tokyo Japan; 2 Department of Social and Preventive Epidemiology, Division of Health Sciences and Nursing, Graduate School of Medicine, The University of Tokyo Tokyo Japan; 3 Department of Nutrition, Dietetics and Food Monash University Melbourne Australia; 4 Nutrition Innovation Centre for Food and Health (NICHE) School of Biomedical Sciences Ulster University Coleraine United Kingdom; 5 Department of Health Communication, School of Public Health, The University of Tokyo Tokyo Japan; 6 Department of Nutritional Epidemiology and Shokuiku National Institutes of Biomedical Innovation, Health and Nutrition Tokyo Japan; 7 Global Health Nursing, Graduate School of Nursing Science St. Luke’s International University Tokyo Japan; 8 Tokyo Foundation for Policy Research Tokyo Japan; 9 Department of Food Function and Labeling, National Institutes of Biomedical Innovation, Health and Nutrition Tokyo Japan; 10 Department of Biostatistics M&D Data Science Center Tokyo Medical and Dental University Tokyo Japan

**Keywords:** diet, nutrition, information, internet, web, Japanese language

## Abstract

**Background:**

The increased availability of content of uncertain integrity obtained through the internet is a major concern. To date, however, there has been no comprehensive scrutiny of the fitness-for-purpose of web-based content on diet and nutrition.

**Objective:**

This cross-sectional study aims to describe diet- and nutrition-related web-based content written in Japanese, identified via a systematic extraction strategy using Google Trends and Google Search.

**Methods:**

We first identified keywords relevant for extracting web-based content (eg, blogs) on diet and nutrition written in Japanese using Google Trends. This process included identification of 638 seed terms, identification of approximately 1500 pairs of *related queries (top)* and *search terms*, the top 10% of which were extracted to identify 160 relevant pairs of *related queries (top)* and *search terms*, and identification of 107 keywords for search. We then extracted relevant web-based content using Google Search.

**Results:**

The content (N=1703) examined here was extracted following a search based on 107 keywords. The most common themes included food and beverages (390/1703, 22.9%), weight management (366/1703, 21.49%), health benefits (261/1703, 15.33%), and healthy eating (235/1703, 13.8%). The main disseminators were information technology companies and mass media (474/1703, 27.83%), food manufacturers (246/1703, 14.45%), other (236/1703, 13.86%), and medical institutions (214/1703, 12.57%). Less than half of the content (790/1703, 46.39%) clearly indicated the involvement of editors or writers. More than half of the content (983/1703, 57.72%) was accompanied by one or more types of advertisement. The proportion of content with any type of citation reference was 40.05% (682/1703). The themes and disseminators of content were significantly associated with the involvement of editors or writers, accompaniment with advertisement, and citation of reference. In particular, content focusing on weight management was more likely to clearly indicate the involvement of editors or writers (212/366, 57.9%) and to be accompanied by advertisement (273/366, 74.6%), but less likely to have references cited (128/366, 35%). Content from medical institutions was less likely to have citation references (62/214, 29%).

**Conclusions:**

This study highlights concerns regarding the authorship, conflicts of interest (advertising), and the scientific credibility of web-based diet- and nutrition-related information written in Japanese. Nutrition professionals and experts should take these findings seriously because exposure to nutritional information that lacks context or seems contradictory can lead to confusion and backlash among consumers. However, more research is needed to draw firm conclusions about the accuracy and quality of web-based diet- and nutrition-related content and whether similar results can be obtained in other major mass media or social media outlets and even other languages.

## Introduction

### Background

Currently, diet- and nutrition-related information is readily available on a range of media platforms. Unfortunately, the credibility of this kind of information is not always guaranteed, which may result in information that should be delivered to the public not being disseminated or, conversely, in the dissemination of information that is not scientifically reliable [1-4]. This presents individuals with significant challenges in evaluating and selecting the sources of information they use and, more importantly, in assessing the credibility and reliability of those sources [4-8]. As a result, the ability of members of the public to maintain and promote their own health may not be best served [9,10]. For example, articles receiving funding from the food industry tend to report greater health benefits for certain foods than other articles [11]. In addition, although numerous meta-analyses have found no clear difference between the weight loss effects of dietary fats and carbohydrates [12,13], contradictory information on this issue is present in the media, which may increase public confusion and distrust in nutritional science [14]. Furthermore, during the COVID-19 pandemic, there was a sharp increase in advertisements for dietary supplements claiming to prevent infection where no scientific evidence exists [15]. Consequently, consumers (especially those with low media literacy and critical evaluation skills) are inundated with web-based information that they cannot adequately scrutinize [16,17].

In the internet age, Google Search is a common tool for discovering web-based information [18], whereas Google Trends is widely used to analyze web-based search behavior and search queries in the field of big data analytics in health care and public health research [19]. However, the increased availability of content of uncertain integrity through the internet is a major cause of concern [20]. To our knowledge, however, previous studies investigating web-based content related to diet and nutrition are limited in terms of topics covered (weight loss, pregnancy, vegan diet, etc) or comprehensiveness [16]. Thus, there has been no comprehensive scrutiny of the fitness-for-purpose of web-based content on diet and nutrition.

### Objective

The aim of this study was to describe diet- and nutrition-related web-based content written in Japanese, identified on the basis of a systematic extraction strategy using Google Trends and Google Search. We hypothesize that the majority of web-based content on diet and nutrition does not clearly indicate editor or writer involvement, is often accompanied by advertising, and lacks cited references.

## Methods

**Figure 1:**
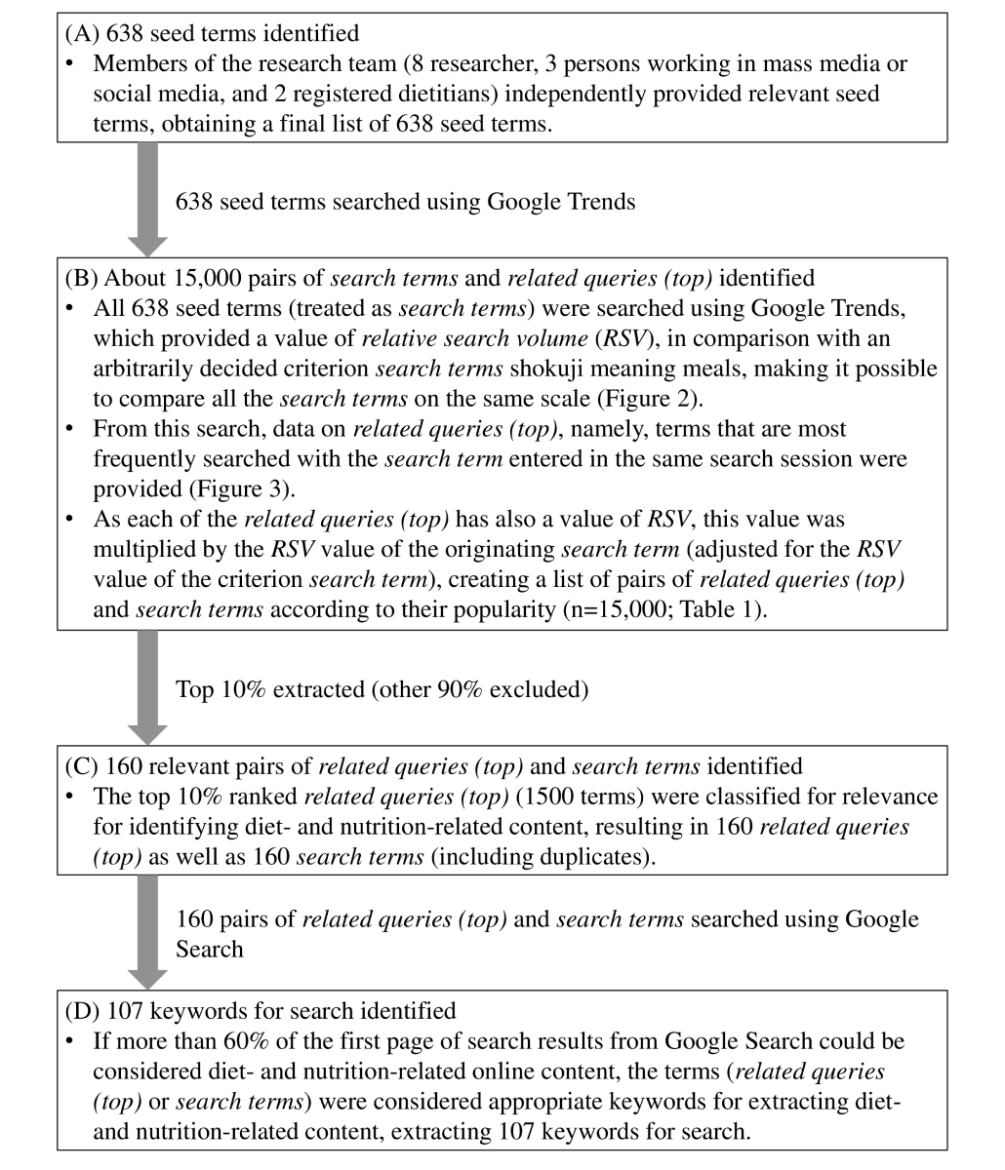
Flowchart of a systematic process for identifying keywords relevant for extracting web-based content on diet and nutrition written in Japanese. (A) identification of 638 seed terms; (B) identification of about 15,000 pairs of search terms and related queries (top); (C) identification of 160 relevant pairs of related queries (top) and search terms; (D) identification of 107 keywords for search.

In this cross-sectional study, we first identified keywords relevant for extracting web-based content (eg, blogs) on diet and nutrition written in Japanese using Google Trends (as described in Figure 1). We then extracted relevant web-based content using Google Search.

### Ethical Considerations

This study did not include human participants, and all data were obtained from the public domain and kept anonymous. Therefore, ethics approval was not required.

### Identification of Keywords Relevant for Extracting Web-Based Content on Diet and Nutrition

#### Identification of Seed Terms to Enter Into Google Trends

Before the search using Google Trends, we determined the initial terminology (ie, seed terms) related to diet and nutrition written in Japanese (Figure 1A). The initial seed terms in Japanese were independently provided by 8 researchers in the fields of nutrition and dietetics, health science, or health communication; 3 persons currently working in mass media or social media with expertise in nutrition and health; and 2 registered dietitians. After removing duplicates, a final list of 638 seed terms was agreed upon (the list of these terms is available from the corresponding author upon request).

#### Identification of Pairs of Search Terms and Related Queries (Top) Using Google Trends

All 638 seed terms were searched using Google Trends. Google Trends is a freely accessible tool dedicated to estimating the *relative search volume* (*RSV*) of queries made in the Google search engine. The *RSV*, an index of search volume adjusted to the number of Google users in a given geographic area and period, ranges from 0 to 100. A value of 100 indicates the peak of popularity (100% popularity in a given period and location), whereas 0 indicates complete disinterest (0%) [19]. The engine enables the analysis of a chosen phrase in a selected region and period (since January 2004). Google Trends allows comparison of up to 5 terms at the same time. In such cases, *RSV* is adjusted, with *RSV*=100 representing the highest popularity of one of the chosen phrases.

**Figure 2:**
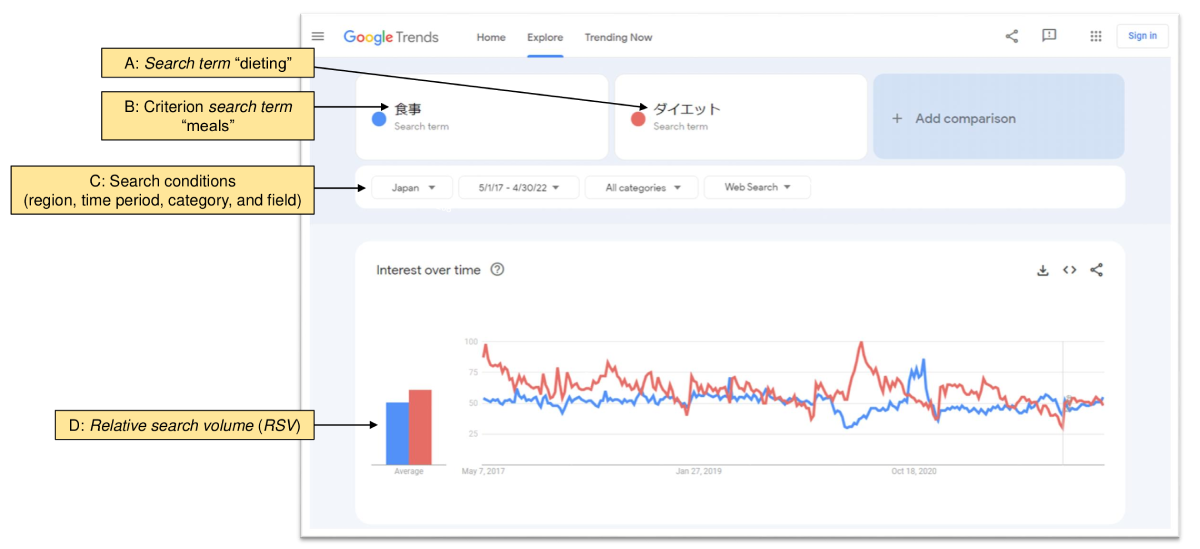
Screenshot of Google Trends as an example. In this study, all 638 seed terms were used as search terms (A and B). We conducted a search for each search term (A: dieting in this example) in comparison with the criterion search term meaning meals (B); in other words, only 2 search terms were compared at once. All searches were carried out under uniform search conditions with regard to region, time period, category, and field (C). By doing this, we obtained the relative search volume (RSV; an index of search volume adjusted to the number of Google users in a given geographic area and period, ranging from 0 to 100; (D) relative to the one criterion search term (B: meals) for all search terms (A: dieting in this example).

Google Trends may qualify the analyzed phrases as *search term* or *topic*. *Search terms* are literally typed words, whereas *topics* may be proposed by Google Trends when the tool recognizes phrases related to popular queries. In this study, all 638 seed terms were used as *search terms* (Figure 1B). As shown in Figure 2, we conducted a search for each *search term* in comparison with the *search term* meaning meals (shokuji in Japanese); in other words, only 2 *search terms* were compared at once. By doing this, we obtained the *RSV* relative to the one criterion *search term* (meals) for all *search terms* so that we could rank all the *search terms* according to *RSV* values (as the *RSV* of each *search term* divided by the *RSV* of the criterion *search term*). All searches were carried out between May 1 and May 31, 2022, under uniform search conditions (Figure 2) with regard to region (Japan), time period (May 1, 2017, to April 30, 2022), category (all categories), and field (web search). We set the search period to be long enough (5 years) and covering not only before but also after the strict COVID-19 policy implementation, which may make people’s behaviors change significantly, to explain the real results.

**Figure 3:**
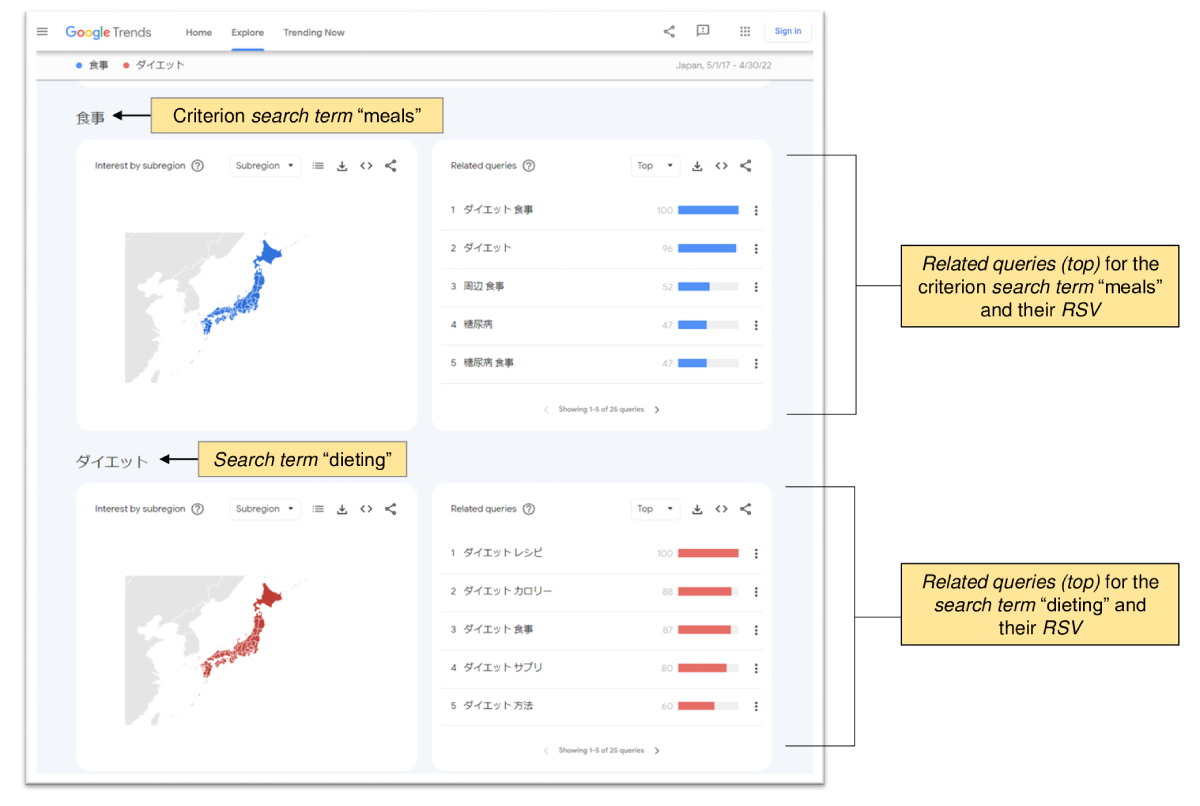
Screenshot of Google Trends as an example. The search conducted using Google Trends (as shown in Figure 2) simultaneously provides data on related queries (top), namely the terms that are most frequently searched with the search term entered in the same search session, within the chosen category, country, or region. As each related query (top) has a value of relative search volume (RSV), this value was multiplied by the RSV value of the originating search term (adjusted for the RSV value of the criterion search term), creating a list of pairs of related queries (top) and search terms according to their popularity.

As shown in Figure 3, the search conducted using Google Trends simultaneously provided data on *related queries (top)*, namely the terms that were most frequently searched with the *search term* entered in the same search session, within the chosen category, country, or region. As each *related query (top)* has a value of *RSV*, this value was multiplied by the *RSV* value of the originating *search term* (adjusted for the *RSV* value of the criterion *search term*), creating a list of pairs of *related queries (top)* and *search terms* according to their popularity (n=approximately 15,000); some examples are shown in Table 1.

**Table 1:** Examples of search terms, related queries (top), and keywords used in our subsequent search^a^.

Search term	RSV^b^ of search term in comparison with the criterion search term “meals”	RSV of the criterion search term “meals”	RSV of search term adjusted for RSV of the criterion search term “meals”	Related query (top)	RSV of related query (top) in comparison with the criterion search term “meals”	Popularity score for related query (top)	Keywords used in our subsequent search
Meals	—^c^	—	1	Dieting Meals	100	100	Dieting Meals
Meals	—	—	1	Dieting	96	96	Dieting
Dieting	60	49	1.224	Dieting Recipe	100	122.4	Dieting Recipe
Dieting	60	49	1.224	Dieting Calories	88	107.7	Dieting Calories
Food	42	57	0.737	Constipation Food	46	33.9	Constipation Food
Food	42	57	0.737	Coronavirus Food	30	22.1	Coronavirus Food

^a^See Figures 2 and 3 for screenshots of Google Trends as examples of search terms and related queries (top), respectively.

^b^RSV: relative search volume.

^c^Not available.

#### Identification of Relevant Pairs of Related Queries (Top) and Search Terms

The top 10% ranked *related queries (top*; n=1500) were independently classified by 2 registered dietitians (NK and HO) by relevance to identify diet- and nutrition-related information (relevant, irrelevant, or unknown). A κ coefficient of 0.93 was obtained between the 2 dietitians, showing a high degree of agreement. After excluding 1340 *related queries (top)* that were classified as irrelevant by both dietitians, 160 *related queries (top)* and the associated 160 *search terms* (including duplicates) were identified (Figure 1C).

#### Identification of the Final Set of “Keywords for Search”

These 160 *related queries (top)* and the associated 160 *search terms* (including duplicates) were searched using Google Search from August 1 to September 2, 2022. All searches were conducted using the privacy or incognito browsing mode, with browser history and cookies cleared [21]. If >60% of the first page of the search results could be considered diet- and nutrition-related information written in Japanese by a registered dietitian (NK or HO), we considered these terms appropriate keywords for extracting diet- and nutrition-related information (Figure 1D). For this, we used the following exclusion criteria, which were mainly informed by a previous study [22] in addition to a pilot study: (1) content not written in Japanese, (2) those only describing animals or cells, (3) those without information on diet or nutrition, (4) those not directed at the public (scientific articles, guidelines, etc), (5) those in which videos were the main content, (6) those in which exchange of opinion was the main content (eg, bulletin board), (7) those in which only recipes were shown, (8) those for which a password and subscription fee were required, (9) those to which access was not possible (eg, page no longer exists), and (10) those that were exclusively shopping sites or advertisements. Consequently, 107 keywords for the search were extracted (the list of these Japanese words is available from the corresponding author upon request).

### Identification of Web-Based Content on Diet and Nutrition Using Google Search

Using the 107 keywords, we extracted nutrition and diet-related web-based content via Google Search for the period August 1 through September 2, 2022. Google accounts for 76.5% of the search engine market share in Japan [23]. As a previous report has shown that 92% of internet users will not click on a website beyond the first 3 pages of the results [2], we decided to examine 3 pages of results for each search. For each piece of content (n=5671) extracted (including duplicates), we checked whether the information was out of scope using the abovementioned exclusion criteria, reducing the sample size to 1703. The variables we assessed and coded were as follows: if ≥1 editors were clearly indicated, if ≥1 authors were clearly indicated, if ≥1 editors or authors were clearly indicated, and if there was accompanying advertisement (ie, some statements within sentences or sidebars and bottom bars for advertisement), title (if any) and clear inclusion of ≥1 references (eg, peer-reviewed articles, nonfiction books, dietary reference intake [DRI] [24], Japanese dish-based dietary guidelines [ie, Food Guide Spinning Top] [25], and other references published by a public organization). Although we did not measure the quality or accuracy of the web-based content, we considered these 3 characteristics (ie, authorship, advertisement, and attribution) as proxies for quality or accuracy [26,27]. Two registered dietitians (NK and HO) performed the coding, with the first (KM) and second (NS) authors setting up the general framework. After pilot coding (n=10 for each dietitian), any ambiguities were identified and discussed by the coding dietitians and the first and second authors until consensus was reached. Because of a lack of sufficient human resources, it was not possible to code in duplicate. Nevertheless, to improve reliability, whenever any further ambiguities were identified during the coding process, a discussion was conducted between the 2 coding dietitians until consensus was reached; if they could not reach consensus, a decision was made by the first author (the list of ambiguities and their solutions or consensus [n=56] is available from the corresponding author upon request).

### Statistical Analysis

All analyses were conducted by the first author using the SAS statistical software (version 9.4; SAS Institute Inc). All data are presented as numbers and percentages. We did not conduct any formal sample size calculations for this descriptive analysis. The content extracted (N=1703) was categorized according to the associated *search terms* into the following themes: food and beverages, weight management, health benefits, healthy eating, nutrition and nutrients, blood and disease, and others. The content was also categorized by disseminators defined as health care and beauty-related companies, food manufacturers, information technology (IT) companies and mass media, government and academic institutions, medical institutions, pharmaceutical manufacturers, and others. Additional characteristics recorded included (1) if the involvement of editors or authors was clearly indicated, (2) if the content was accompanied by advertisement, and (3) whether the content included a citation or citations from peer-reviewed articles, nonfiction books, DRI, Japanese dish-based dietary guidelines, and other materials published by public organizations. Finally, the associations of these characteristics for the content with the themes or disseminators of the content were examined using the chi-square test, with a 2-tailed *P* value of <.05 considered significant.

## Results

### Common Themes in Web-Based Content on Diet and Nutrition

**Figure 4:**
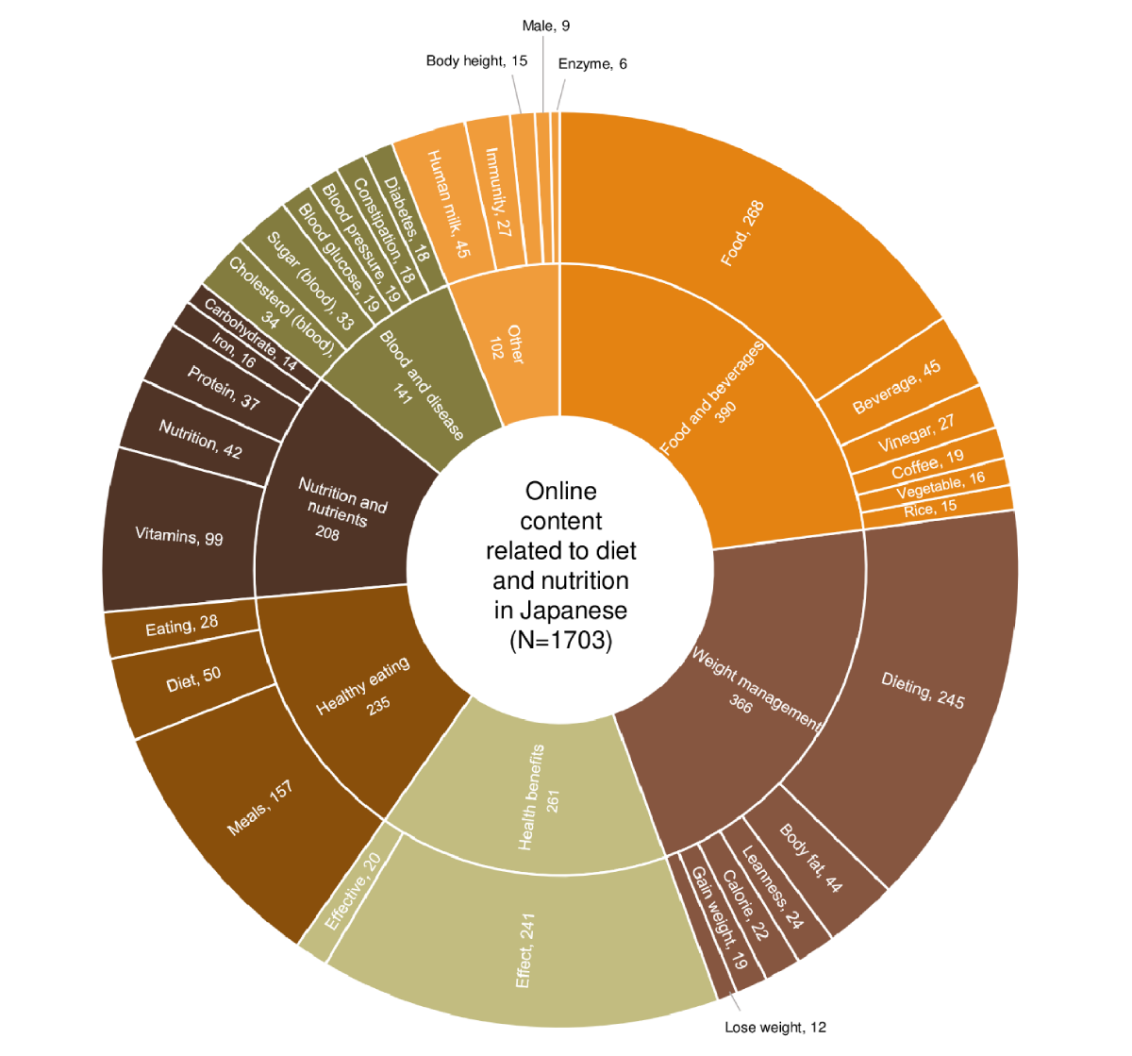
Search terms (outer layer) used to identify web-based content related to diet and nutrition in Japanese and the topic themes (inner layer). The number of web-based content is shown (N=1703).

The content (N=1703) examined here was extracted following a search based on 107 keywords, consisting of *related queries (top)* and *search terms*. As shown in Figure 4, the most efficient keywords (at the level of *search terms*) included “food” (268/1703, 15.74%), “dieting” (245/1703, 14.39%), “effect” (241/1703, 14.15%), and “meals” (157/1703, 9.22%); these together identified more than half of the content (911/1703, 53.49%). In contrast, each of the 29 remaining keywords (at the level of *search terms*) identified <6% of the content. On the basis of these 33 keywords (at the level of *search terms*), the content was classified according to the themes (Figure 4). The most dominant theme was food and beverages (390/1703, 22.9%), followed in order by weight management (366/1703, 21.49%), health benefits (261/1703, 15.33%), healthy eating (235/1703, 13.8%), nutrition and nutrients (208/1703, 12.21%), blood and disease (141/1703, 8.28%), and other (102/1703, 5.99%).

### Disseminators of Web-Based Content on Diet and Nutrition

**Figure 5:**
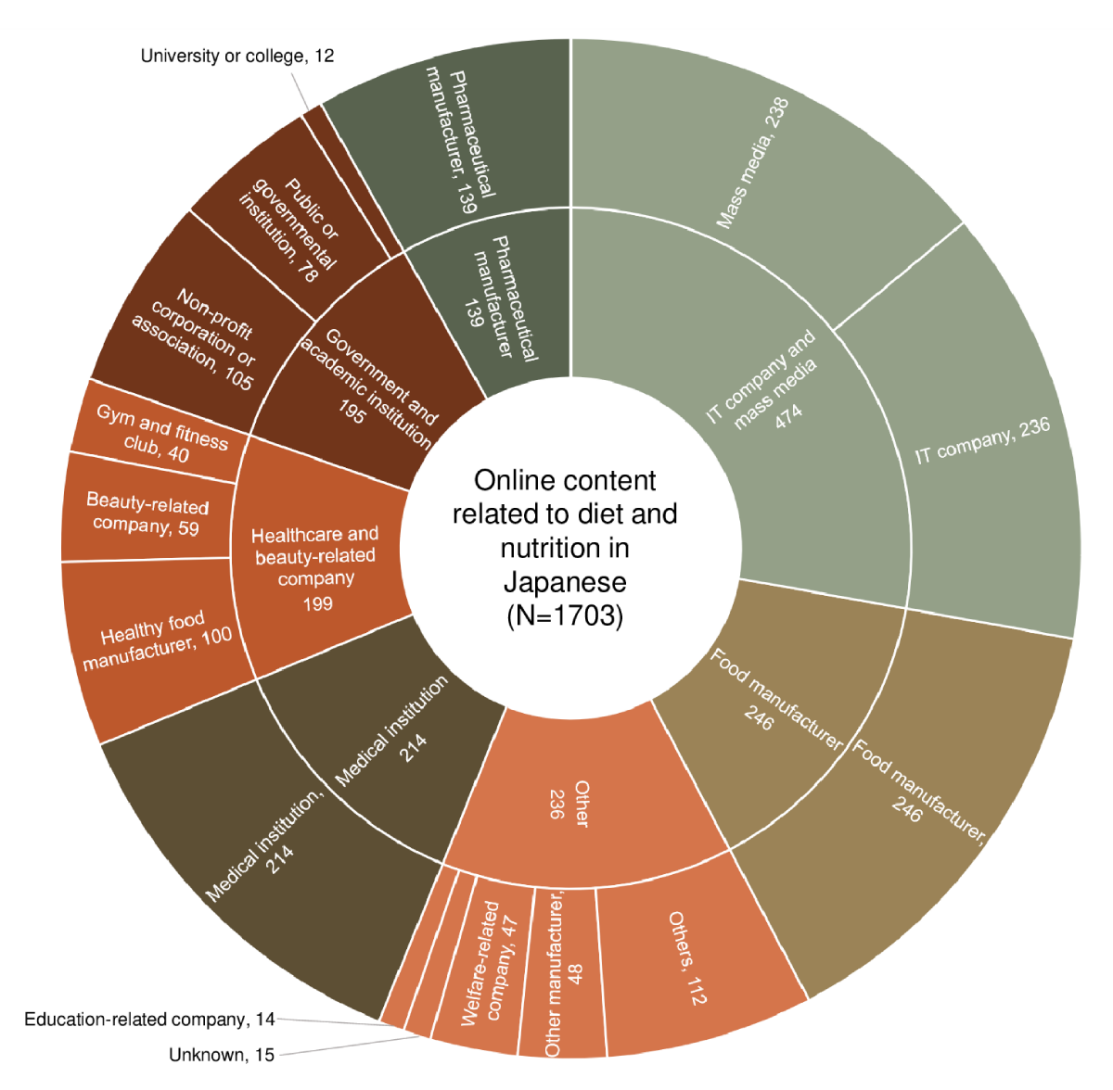
Disseminators (outer layer) and their categories (inner layer) of web-based content related to diet and nutrition written in Japanese. The number of web-based content is shown (N=1703).

As shown in Figure 5, the key disseminators of web-based content were IT companies and mass media (474/1703, 27.83%), followed in order by food manufacturers (246/1703, 14.45%), others (236/1703, 13.86%), medical institutions (214/1703, 12.57%), health care and beauty-related companies (199/1703, 11.69%), government and academic institutions (195/1703, 11.45%), and pharmaceutical manufacturers (139/1703, 8.16%).

### Additional Characteristics of Web-Based Content on Diet and Nutrition

Less than half of the content clearly indicated the involvement of editors (562/1703, 33%), writers (321/1703, 18.85%), and editors or writers (790/1703, 46.39%; Table 2). In contrast, more than half of the content was accompanied by some form of advertisement (983/1703, 57.72%). Furthermore, only a small proportion of content clearly cited references: 10.22% (174/1703) for scientific papers, 7.93% (135/1703) for nonfiction books, 17.67% (301/1703) for DRIs, 3.76% (64/1703) for the Japanese dish-based dietary guidelines, and 22.02% (375/1703) for other references published by public organizations. The proportion of content with any type of citation reference was only 40.05% (682/1703).

**Table 2:** Associations between themes and characteristics of web-based content on diet and nutrition written in Japanese (N=1703).

		All (N=1703), n (%)	Food and beverages (n=390), n (%)	Weight management (n=366), n (%)	Health benefits (n=261), n (%)	Healthy eating (n=235), n (%)	Nutrition and nutrients (n=208), n (%)	Blood and disease (n=141), n (%)	Other (n=102), n (%)	*P* value^a^
**Involvement of editors indicated**										
	No	1141 (67)	236 (60.5)	225 (61.5)	178 (68.2)	182 (77.4)	172 (82.7)	82 (58.2)	66 (64.7)	<.001
	Yes	562 (33)	154 (39.9)	141 (38.5)	83 (31.8)	53 (22.6)	36 (17.3)	59 (41.8)	36 (35.3)	<.001
**Involvement of writers indicated**										
	No	1382 (81.2)	324 (83.1)	257 (70.2)	204 (78.2)	202 (86)	190 (91.3)	117 (83)	88 (86.3)	<.001
	Yes	321 (18.8)	66 (16.9)	109 (29.8)	57 (21.8)	33 (14)	18 (8.7)	24 (17)	14 (13.7)	<.001
**Involvement of editors or writers indicated**										
	No	913 (53.6)	183 (46.9)	154 (42.1)	145 (55.6)	156 (66.4)	155 (74.5)	64 (45.4)	56 (54.9)	<.001
	Yes	790 (46.4)	207 (53.1)	212 (57.9)	116 (44.4)	79 (33.6)	53 (25.5)	77 (54.6)	46 (45.1)	<.001
**Advertisement included (ie, some statements within sentences or sidebars and bottom bars for advertisement)**										
	No	720 (42.3)	150 (38.5)	93 (25.4)	89 (34.1)	141 (60)	112 (53.8)	89 (63.1)	46 (45.1)	<.001
	Yes	983 (57.7)	240 (61.5)	273 (74.6)	172 (65.9)	94 (40)	96 (46.2)	52 (36.9)	56 (54.9)	<.001
**Scientific papers cited**										
	No	1529 (89.8)	355 (91)	334 (91.3)	222 (85.1)	220 (93.6)	180 (86.5)	129 (91.5)	89 (87.3)	.02
	Yes	174 (10.2)	35 (9)	32 (8.7)	39 (14.9)	15 (6.4)	28 (13.5)	12 (8.5)	13 (12.7)	.02
**Nonfiction books cited**										
	No	1568 (92.1)	371 (95.1)	340 (92.9)	240 (92)	213 (90.6)	180 (86.5)	135 (95.7)	89 (87.3)	.002
	Yes	135 (7.9)	19 (4.9)	26 (7.1)	21 (8)	22 (9.4)	28 (13.5)	6 (4.3)	13 (12.7)	.002
**Dietary reference intakes cited**										
	No	1402 (82.3)	323 (82.8)	305 (83.3)	223 (85.4)	215 (91.5)	124 (59.6)	117 (83)	95 (93.1)	<.001
	Yes	301 (17.7)	67 (17.2)	61 (16.7)	38 (14.6)	20 (8.5)	84 (40.4)	24 (17)	7 (6.9)	<.001
**Dish-based dietary guidelines cited**										
	No	1639 (96.2)	381 (97.7)	353 (96.4)	260 (99.6)	206 (87.7)	203 (97.6)	139 (98.6)	97 (95.1)	<.001
	Yes	64 (3.8)	9 (2.3)	13 (3.6)	1 (0.4)	29 (12.3)	5 (2.4)	2 (1.4)	5 (4.9)	<.001
**Other materials published by public organizations cited**										
	No	1328 (78)	302 (77.4)	307 (83.9)	214 (82)	177 (75.3)	141 (67.8)	105 (74.5)	82 (80.4)	<.001
	Yes	375 (22)	88 (22.6)	59 (16.1)	47 (18)	58 (24.7)	67 (32.2)	36 (25.5)	20 (19.4)	<.001
**At least one of the above references (ie, scientific papers, nonfiction books, dietary reference intakes, dish-based dietary guidelines, or other materials published by public organizations) cited**										
	No	1021 (60)	250 (64.1)	238 (65)	159 (60.9)	139 (59.1)	87 (41.8)	86 (61)	62 (60.8)	<.001
	Yes	682 (40)	140 (35.9)	128 (35)	102 (39.1)	96 9 (40.9)	121 (58.2)	55 (39)	40 (39.2)	<.001

^a^Using chi-square test.

### Associations Between the Theme and Characteristics of Web-Based Content on Diet and Nutrition

All the associations between the themes and characteristics of web-based content on diet and nutrition were statistically significant according to the chi-square test (Table 2). The content with the theme of nutrition and nutrients was less likely to clearly indicate the involvement of editors (36/208, 17.3%), writers (18/208, 8.7%), or either editors or writers (53/208, 25.5%) compared with that with other themes, particularly blood and disease (59/141, 41.8% for editors) and weight management (109/366, 29.8% for writers and 212/366, 57.9% for either editors or writers). The likelihood of being accompanied by any kind of advertisement was highest for content focusing on weight management (273/366, 74.6%) and lowest for content focusing on blood and disease (52/141, 36.9%). For citing references, the results varied depending on the source of citations, but content focusing on nutrition and nutrients generally cited at least some references (28/208, 13.5% for nonfiction books; 84/208, 40.4% for DRIs; and 67/208, 32.2% for other references) compared with those focusing on other themes. When all citation sources were considered collectively, the likelihood that references were cited was highest in content focusing on nutrition and nutrients (121/208, 58.2%) and lowest in content focusing on weight management (128/366, 35%).

### Associations Between Disseminator and Characteristics of Web-Based Content on Diet and Nutrition

Associations between disseminator and characteristics of web-based content on diet and nutrition are shown in Table 3. The content provided by IT companies and mass media was more likely to clearly indicate the involvement of editors (246/474, 51.9%), writers (200/474, 42.2%), or either editors or writers (367/474, 77.4%) compared with that derived from other disseminators, particularly government and academic institutions (14/195, 7.2% for editors and 34/195, 17.4% for either editors or writers) and pharmaceutical manufacturers (7/139, 5% for writers). Accompanying advertisements were highest in content from IT companies and mass media (446/474, 94.1%) and lowest in content from government and academic institutions (5/195, 2.6%). For citing references, the results varied depending on the source of citations (with no significant associations for peer-reviewed articles and nonfiction books), but content from government and academic institutions generally tended to have at least some references (26/195, 13.3% for the Japanese dish-based dietary guidelines and 60/195, 30.8% for other citation materials) compared with that from other disseminators. When the citation references were considered collectively, the likelihood that references were cited was highest for content from government and academic institutions (111/195, 56.9%) and lowest for content from medical institutions (62/214, 29%).

**Table 3:** Associations between disseminators and characteristics of web-based content on diet and nutrition written in Japanese (N=1703).

Characteristics			Health care– and beauty-related company (n=199), n (%)		Food manufacturer (n=246), n (%)		IT company and mass media (n=474), n (%)		Government and academic institution (n=195), n (%)		Medical institution (n=214), n (%)		Pharmaceutical manufacturer (n=139), n (%)		Other (n=236), n (%)		*P* value^a^
**Involvement of editors indicated**																	
	No	132 (66.3)		201 (81.7)		228 (48.1)		181 (92.8)		167 (78)		77 (55.4)		155 (65.7)		<.001	
	Yes	67 (33.7)		45 (18.3)		246 (51.9)		14 (7.2)		47 (22)		62 (44.6)		81 (34.3)		<.001	
**Involvement of writers indicated**																	
	No	179 (89.9)		228 (92.7)		274 (57.8)		173 (88.7)		176 (82.2)		132 (95)		220 (93.2)		<.001	
	Yes	20 (10.1)		18 (7.3)		200 (42.2)		22 (11.3)		38 (17.8)		7 (5)		16 (6.8)		<.001	
**Involvement of editors or writers indicated**																	
	No	114 (57.3)		184 (74.8)		107 (22.6)		161 (82.6)		134 (62.6)		70 (50.4)		143 (60.6)		<.001	
	Yes	85 (42.7)		62 (25.2)		367 (77.4)		34 (17.4)		80 (37.4)		69 (49.6)		93 (39.4)		<.001	
**Advertisement included (ie, some statements within sentences or sidebars and bottom bars for advertisement)**																	
	No	70 (35.2)		90 (36.6)		28 (5.9)		190 (97.4)		181 (84.6)		79 (56.8)		82 (34.7)		<.001	
	Yes	129 (64.8)		156 (63.4)		446 (94.1)		5 (2.6)		33 (15.4)		60 (43.2)		154 (65.3)		<.001	
**Scientific papers cited**																	
	No	179 (89.9)		215 (87.4)		433 (91.4)		166 (85.1)		199 (93)		129 (92.9)		208 (88.1)		.06	
	Yes	20 (10.1)		31 (12.6)		41 (8.6)		29 (14.9)		15 (7)		10 (7.2)		28 (11.9)		.06	
**Nonfiction books cited**																	
	No	181 (91)		225 (91.5)		439 (92.6)		174 (89.2)		199 (93)		129 (92.8)		221 (93.6)		.68	
	Yes	18 (9)		21 (8.5)		35 (7.4)		21 (10.8)		15 (7)		10 (7.2)		15 (6.4)		.68	
**Dietary reference intakes cited**																	
	No	162 (81.4)		175 (71.1)		430 (90.7)		150 (76.9)		187 (87.4)		108 (77.7)		190 (80.5)		<.001	
	Yes	37 (18.6)		71 (28.9)		44 (9.3)		45 (23.1)		27 (12.6)		31 (22.3)		46 (19.5)		<.001	
**Dish-based dietary guidelines cited**																	
	No	193 (97)		231 (93.9)		472 (99.6)		169 (86.7)		209 (97.7)		138 (99.3)		227 (96.2)		<.001	
	Yes	6 (3)		15 (6.1)		2 (0.4)		26 (13.3)		5 (2.3)		1 (0.7)		9 (3.8)		<.001	
**Other materials published by public organizations cited**																	
	No	159 (79.9)		183 (74.4)		379 (80)		135 (69.2)		184 (86)		107 (77)		181 (76.7)		.002	
	Yes	40 (20.1)		63 (25.6)		95 (20)		60 (30.8)		30 (14)		32 (23)		55 (23.3)		.002	
**At least one of the above references (ie, scientific papers, nonfiction books, dietary reference intakes, dish-based dietary guidelines, or other materials published by public organizations) cited**																	
	No	118 (59.3)		136 (55.3)		312 (65.8)		84 (43.1)		152 (71)		82 (59)		137 (58.1)		<.001	
	Yes	81 (40.7)		110 (44.7)		162 (34.2)		111 (56.9)		62 (29)		57 (41)		99 (41.9)		<.001	

^a^Using chi-square test.

## Discussion

### Principal Findings

This study provides a comprehensive picture of web-based diet and nutrition information in Japanese. Content was extracted through a systematic process based on Google Trends and Google Search. The top 5 themes were food and beverages, weight management, health benefits, healthy eating, and nutrition and nutrients, which accounted for 85.73% (1460/1703) of the total. Meanwhile, the top 5 disseminators were IT companies and mass media, food manufacturers, others, medical institutions, and health care and beauty-related companies, which accounted for 80.39% (1369/1703) of the total. Only 46.39% (790/1703) of the content had a clear editor or author and 57.72% (983/1703) of the content accompanied some form of advertising. In addition, only 40.05% (682/1703) of the studies cited some type of literature. The themes and disseminators of the content were significantly related to the characteristics of the content. In particular, content on the theme of weight management was more likely to clearly identify an editor or author (212/366, 57.9%) and to be accompanied by some form of advertising (273/366, 74.6%), whereas they were less likely to cite any kind of literature (128/366, 35%). In addition, content from medical institutions was less likely to cite any type of literature (62/214, 29%). To our knowledge, this is the first study to provide a comprehensive overview of the sources and characteristics of web-based content on diet and nutrition.

### Comparison With Prior Work

A previous analysis of nutrition-related blog posts found that the predominant theme was dietary recommendations, with a particular focus on increasing intake of fruit and vegetable [28]. In addition, an analysis of blogs identified calorie counting and diet restriction as the top 2 dominant themes [29]. In contrast, our approach identified a much wider range of themes in web-based diet- and nutrition-related content in Japanese. Nevertheless, the present findings should be interpreted in light of the demographics and behaviors of those seeking web-based content. A nationwide survey in Japan, for example, found that the younger generation (aged 20-40 years) spent more time using the internet than watching television, but the opposite was true for the older generation (aged 50-69 years) [30]. Another nationwide survey suggested that television may be a more dominant source of dietary information, particularly in older adults [31]. Thus, it is possible that themes popular with older adults were not adequately covered in this study. Future research based on information provided by other media (especially television), on which the older adults rely heavily, would be of great value.

We found that diet- and nutrition-related web-based content was provided by a variety of sources, namely companies and institutions in Japan. A limited number of studies have shown that a variety of companies and institutions disseminate web-based information on, for example, bariatric surgery [32], testosterone supplementation [33], and autism [26]. Taken together, our findings suggest that the comprehensiveness of the method we used to extract web-based content, rather than just keywords, may be applicable to future research on more specific themes, information disseminators, and other media (eg, YouTube).

In this study, editors and authors were not clearly identified in approximately half of the content. An analysis of national daily newspapers from the United Kingdom showed that the quality scores of anonymous health-related articles were significantly lower than those attributed to named journalists [34]. We also found that more than half of the web-based content included some form of advertisement. Certainly, the absence of advertising does not necessarily reflect credibility [29], but the inherent conflict of interest adduced to advertising is of concern because content can be manipulated to provide information that would be favorable to advertising [35]. Furthermore, two-fifths of the content in this study did not cite any reference. Moreover, although the presence of references does not necessarily guarantee reliability, it is clear that content that is not evidence-based can only result in confusion to the public at best [1]. Overall, our findings are consistent with a limited number of studies that found that the quality or accuracy of web-based content on diet and nutrition was generally problematic [20,22,29,36]. Although more data are needed, these findings may help determine whether web-based content about diet and nutrition should be subject to regulation or verification of credibility [29]. Another potential solution may include improving the eHealth and media literacy of consumers [16]. In addition, to counteract misinformation, it may also be imperative for nutrition professionals and experts to publish accurate and high-quality, web-based nutrition information, by carefully avoiding common mistakes, including the omission of reference to original source material [16].

Interestingly, we found significant associations between the themes and disseminators and characteristics of web-based content on diet and nutrition. In particular, content on the theme of weight management tended to clearly indicate the involvement of editors or authors while tending to be accompanied by some kind of advertising and without citation of references. This might suggest that authors with some authority (eg, registered dietitians) disseminate information that has no scientific basis but is convenient for the promotion of a particular product or service on a theme of interest to the public [28]. Regarding disseminators of web-based content, we found that the likelihood of citing references was lowest in content from medical institutions (62/214, 29%). The reason for this is unknown, but such information may be an expression of food-related philosophy [29] or daily activities (such as family cooking) [28]. Ultimately, these phenomena might be attributable to the inadequate nature of training courses, research centers, and academia in the field of public health nutrition in Japan [37]. Therefore, empirical data on this point are urgently needed.

### Limitations

This study had several limitations. First, the initial terminology (seed terms) for the searches based on Google Trends was determined using a snowball process within the research team. This is subjective and would have yielded a variety of different term sequences if performed by different teams. However, to obtain as broad a range of seed terms as possible, we enlisted the help of persons active on mass media and social media and registered dietitians. The keywords for search were finalized based on Google Trends, which is considered to reflect the actual keywords for search used by the public. Nevertheless, the Google Ads Keywords Planner, which provides a list of relevant terms for search and their search volume for the last 48 months after entering a particular word or phrase [38], might have been a more suitable choice for the selection of keywords for search, and thus should be considered for future research. Second, it should be noted that searches based on Google Search are not reproducible because of the search algorithms’ dynamic and unknown nature [21,39]. In addition, the web-based environment changes rapidly, and this study provides only a “snapshot” in time. Thus, the cross-sectional nature of this study is a significant limitation. Furthermore, the use of Google Trends and Google Search may have biased the results toward a certain demographic that used these platforms more frequently. The present findings should be carefully interpreted in this context. Third, coding of the web-based content was performed by 2 registered dietitians without pure double-checking. Although they have expertise in food, diet, nutrition, and cooking, we cannot rule out the possibility of biased coding, coding errors, or both. To minimize such errors, however, ambiguities identified during coding were resolved through discussion between the 2 coding dietitians and, whenever needed, with the first author. Fourth, we did not evaluate the accuracy or quality of the diet- and nutrition-related information extracted in this study mainly because the large number of disseminators and wide range of themes made verification using a uniform procedure impossible. Therefore, we plan to examine the accuracy and quality of the web-based content after carefully selecting relevant themes in future research. Fifth, this study does not allow us to determine who is seeking web-based content on diet and nutrition or what types of web-based content are most influential. In fact, evidence is largely limited with regard to, for example, how popular the use of web-based search is to guide people’s diet- and nutrition-related behavior, to what extent the public relies on or uses the web-based information for such a purpose, and what will be the health consequences if the public gets incorrect information or is misled by media; these questions need to be investigated in future studies. Sixth, this study only included content obtained via the internet and did not include information from other major mass media (television, radio, magazines, etc) or social media (Twitter, Instagram, YouTube, etc). Therefore, whether the present findings are specific to web-based content awaits further research. Similarly, it is unknown whether the present findings based on web-based content in Japanese can be applied to content in other languages. This question is beyond the scope of this study and should be investigated in future research. The final limitation is that this analysis was conducted manually and therefore only included a small portion but highly viewed portion of the content related to diet and nutrition. Application of data science techniques in collaboration with nutrition and dietetics professionals will permit for a larger sample size and the potential for verification of the credibility of the information.

### Conclusions

In conclusion, this study suggests concerns regarding the authorship, conflicts of interest (advertising), and scientific credibility of web-based diet- and nutrition-related information written in Japanese. Nutrition professionals and experts should take these findings seriously because exposure to nutritional information that lacks context or seems contradictory can lead to confusion and backlash among consumers [9,14]. However, more research is needed to draw firm conclusions about the accuracy and quality of web-based diet- and nutrition-related content and whether similar results can be obtained in other major mass media, social media, and even other languages. As a first step, we are currently using the information from this study to conduct several studies focusing on popular topics in web-based content related to diet and nutrition. These include a Twitter content analysis on nutrition and hypertension and an assessment of the quality and reliability of YouTube videos (both in Japanese and English) on nutrition type 2 diabetes, which we believe will significantly help guide the development of new consumer-oriented resources.

## Abbreviations

DRI: dietary reference intake
RSV: relative search volume

## Data Availability

The data sets used or analyzed in this study are available from the corresponding author upon reasonable request.

## References

[1] Mete R, Kellett J, Bacon R, Shield A, Murray K (2021). The P.O.S.T guidelines for nutrition blogs: a modified e-Delphi study. J Acad Nutr Diet.

[2] Eysenbach G, Köhler C (2002). How do consumers search for and appraise health information on the world wide web? Qualitative study using focus groups, usability tests, and in-depth interviews. BMJ.

[3] Fergie G, Hunt K, Hilton S (2013). What young people want from health-related online resources: a focus group study. J Youth Stud.

[4] Sbaffi L, Rowley J (2017). Trust and credibility in web-based health information: a review and agenda for future research. J Med Internet Res.

[5] Metzger MJ, Flanagin AJ (2013). Credibility and trust of information in online environments: the use of cognitive heuristics. J Pragmat.

[6] Gray NJ, Klein JD, Noyce PR, Sesselberg TS, Cantrill JA (2005). The internet: a window on adolescent health literacy. J Adolesc Health.

[7] Gray NJ, Klein JD, Noyce PR, Sesselberg TS, Cantrill JA (2005). Health information-seeking behaviour in adolescence: the place of the internet. Soc Sci Med.

[8] Corritore CL, Wiedenbeck S, Kracher B, Marble RP (2012). Online trust and health information websites. Int J Technol Hum Interact.

[9] Nagler RH (2014). Adverse outcomes associated with media exposure to contradictory nutrition messages. J Health Commun.

[10] Lee CJ, Nagler RH, Wang N (2018). Source-specific exposure to contradictory nutrition information: documenting prevalence and effects on adverse cognitive and behavioral outcomes. Health Commun.

[11] Massougbodji J, Le Bodo Y, Fratu R, De Wals P (2014). Reviews examining sugar-sweetened beverages and body weight: correlates of their quality and conclusions. Am J Clin Nutr.

[12] Naude CE, Schoonees A, Senekal M, Young T, Garner P, Volmink J (2014). Low carbohydrate versus isoenergetic balanced diets for reducing weight and cardiovascular risk: a systematic review and meta-analysis. PLoS One.

[13] Johnston BC, Kanters S, Bandayrel K, Wu P, Naji F, Siemieniuk RA, Ball GD, Busse JW, Thorlund K, Guyatt G, Jansen JP, Mills EJ (2014). Comparison of weight loss among named diet programs in overweight and obese adults: a meta-analysis. JAMA.

[14] Clark D, Nagler RH, Niederdeppe J (2019). Confusion and nutritional backlash from news media exposure to contradictory information about carbohydrates and dietary fats. Public Health Nutr.

[15] Okuhara T, Yokota R, Shirabe R, Iye R, Okada H, Kiuchi T, Chiba T, Akamatsu R (2021). Japanese newspaper advertisements for dietary supplements before and after COVID-19: a content analysis. BMJ Open.

[16] Denniss E, Lindberg R, McNaughton SA (2023). Quality and accuracy of online nutrition-related information: a systematic review of content analysis studies. Public Health Nutr.

[17] Rubin VL (2019). Disinformation and misinformation triangle: a conceptual model for “fake news” epidemic, causal factors and interventions. J Doc.

[18] Brin S, Page L (2012). Reprint of: the anatomy of a large-scale hypertextual web search engine. Comput Netw.

[19] Nuti SV, Wayda B, Ranasinghe I, Wang S, Dreyer RP, Chen SI, Murugiah K (2014). The use of google trends in health care research: a systematic review. PLoS One.

[20] Le L, Finn A (2016). Evaluating credibility of online nutrition information: a content analysis on current nutrition-related blogs. J Acad Nutr Diet.

[21] Cai HC, King LE, Dwyer JT (2021). Using the Google™ search engine for health information: is there a problem? Case study: supplements for cancer. Curr Dev Nutr.

[22] Lambert K, Mullan J, Mansfield K, Koukomous A, Mesiti L (2017). Evaluation of the quality and health literacy demand of online renal diet information. J Hum Nutr Diet.

[23] (2022). Search engine market share Japan. Statcounter.

[24] (2020). Report of the study group on the formulation of the "dietary reference intakes for Japan people". Ministry of Health, Labour and Welfare, Japan.

[25] (2005). Report of the food guide study group: food guide spinning top. Food Guide Study Group, The Ministry of Health, Labour and Welfare and the Ministry of Agriculture, Forestry and Fisheries.

[26] Reichow B, Halpern JI, Steinhoff TB, Letsinger N, Naples A, Volkmar FR (2012). Characteristics and quality of autism websites. J Autism Dev Disord.

[27] Robillard JM, Jun JH, Lai J, Feng TL (2018). The QUEST for quality online health information: validation of a short quantitative tool. BMC Med Inform Decis Mak.

[28] Chan T, Drake T, Vollmer RL (2020). A qualitative research study comparing nutrition advice communicated by registered dietitian and non-registered dietitian bloggers. J Commun Healthc.

[29] Sabbagh C, Boyland E, Hankey C, Parrett A (2020). Analysing credibility of UK social media influencers' weight-management blogs: a pilot study. Int J Environ Res Public Health.

[30] (2022). Survey on information and communication media usage time and information behavior. Institute for Information and Communications Policy, Ministry of Internal Affairs and Communications.

[31] The national health and nutrition survey in Japan. Ministry of Health, Labour and Welfare, Japan.

[32] Barajas-Gamboa JS, Klingler M, Landreneau J, Strong A, Al Zubaidi A, Sharadgah H, Del Gobbo GD, Abril C, Kroh M, Corcelles R (2020). Quality of information about bariatric surgery on the internet: a two-continent comparison of website content. Obes Surg.

[33] Sehn E, Mozak C, Yuksel N, Sadowski CA (2019). An analysis of online content related to testosterone supplementation. Aging Male.

[34] Robinson A, Coutinho A, Bryden A, McKee M (2013). Analysis of health stories in daily newspapers in the UK. Public Health.

[35] Mandoh M, Curtain C (2017). Quality of claims and references found in Australian pharmacy journal advertisements. Int J Pharm Pract.

[36] Hirasawa R, Saito K, Yachi Y, Ibe Y, Kodama S, Asumi M, Horikawa C, Saito A, Heianza Y, Kondo K, Shimano H, Sone H (2012). Quality of internet information related to the Mediterranean diet. Public Health Nutr.

[37] Shinozaki N, Wang HC, Yuan X, Li T, Asano K, Kobayashi S, Sasaki S (2019). Current status of education and research on public health nutrition in Japan: comparison with South Korea, Taiwan, and mainland China. BMC Nutr.

[38] Kain A, Tizek L, Wecker H, Wallnöfer F, Biedermann T, Zink A (2023). Evaluating public interest in herpes zoster in Germany by leveraging the internet: a retrospective search data analysis. BMC Public Health.

[39] Siddhanamatha HR, Heung E, Lopez-Olivo ML, Abdel-Wahab N, Ojeda-Prias A, Willcockson I, Leong A, Suarez-Almazor ME (2017). Quality assessment of websites providing educational content for patients with rheumatoid arthritis. Semin Arthritis Rheum.

